# Impact of Competing Values and Choices on Democratic Support in Hong Kong

**DOI:** 10.1007/s11205-012-0090-0

**Published:** 2012-06-09

**Authors:** Wai-man Lam

**Affiliations:** Department of Politics and Public Administration, The University of Hong Kong, Pokfulam Road, Hong Kong

**Keywords:** Political values, Indicators of democratic support, Democratic consolidation, Hong Kong

## Abstract

This paper examines the reasons for the relatively low democratic support (DS) in Hong Kong in the context of competing values and choices based on the previous Asian Barometer Surveys. In so doing, it establishes a three-factor theoretical model that includes survey attitudinal statements related to authoritarianism (AU), nationalism (NA) and economic evaluations (EC) on DS. Using confirmatory factor analysis (CFA), the analysis shows that the hypothesized model is a very good fit. The Hong Kong people’s relatively low DS, in terms of their unconditional support for democracy and the degree of democracy they want for Hong Kong, can be well explained by the three factors in combination. The factors have various extent of impact on DS, with AU being the strongest, followed by EC, and then NA. The paper contributes by illustrating the usefulness of CFA in political values research, unraveling the comparative importance of the values and choices in affecting DS, and establishing a model for further testing.

## Introduction

Despite of the democratic and economic development in the East Asian region in recent decades, unconditional support for democracy by the local population in the region has remained relatively low. Hong Kong, with its truncated histories of being a British colony and a Chinese city after political resumption, has exhibited almost the least unconditional democratic support among the East Asian countries, disregarding that many of the locals also want their city to be more democratic. This paper examines the reasons for the relatively low democratic support in Hong Kong in the light of competing values and choices, based on the findings of previous Asian Barometer Surveys (ABS).^1^ In so doing, it attempts to establish a three-factor theoretical model that sheds light on the impact of economic evaluations (EC), authoritarianism (AU), and nationalism (NA) in combination on democratic support (DS). Using confirmatory factor analysis (CFA) of LISREL, a method to estimate and fit a hypothesized model for the variables, the paper analyzes the relationships of a wide spectrum of factors/variables with DS in Hong Kong, based on the 2007 ABS Hong Kong findings. Empirically, it argues that Hong Kong people’s support for democracy was affected by values and choices highlighted in the analysis; more importantly by those related to first, AU, and second, EC, but not so much to those about NA. Theoretically, the model established in the paper can also be further tested and applied in analyzing DS in other transitional societies and other periods of Hong Kong. Methodologically, it contributes by illustrating the usefulness of CFA in political values research.

## A Semi-democracy—Democratic Support in Hong Kong

Hong Kong has remained a semi-democracy before and after political resumption, despite having achieved the prerequisite socio-economic conditions of democratization from the 1970s onwards. Over the years, demands for universal suffrage in the elections of the Legislative Council (LegCo) and the Chief Executive (CE) of Hong Kong have never waned. The Basic Law, the mini-constitution of Hong Kong promulgated upon political resumption, promised democracy in a gradual manner. As of 2012, the CE would be elected by an Election Committee composed of 1,200 members returned by limited and selected sectors of the community (membership of the Election Committee was only 800 in the last CE election). While for the LegCo, half of the 70 members would be returned by five geographical constituencies (GC) of around 3.4 million registered voters on universal suffrage, and the other 30 members by 28 functional constituencies (FC) of around 230,000 registered voters. The remaining five members would be nominated by elected District Councilors, and elected by all registered voters who currently do not have a right to vote in FC elections. FC’s eligible voters include designated individuals and legal entities such as organizations and corporations, representing predominantly business and professional interests.

Over the decades, the non-uniform election methods within the FC, the unfair nature of their representation, and the institutionalized constraints FC placed on limiting the power and mitigating the influence of the GC-returned legislators have attracted lots of criticisms and demands for their abolition. While the limited representation and eligibility of the election of the CE has aroused general suspicion of interference, if not manipulation, from China ever since the political resumption in 1997. Amidst the political debate on all these questions, the Standing Committee of the National People’s Congress of China ruled on Hong Kong’s constitutional development in 2004 that the ratio between LegCo members returned by GC and FC elections shall be half and half, meaning that FC elections shall not be abolished in the foreseeable future. In 2007, the Standing Committee ruled that the electoral system of Hong Kong shall be further, not fully, democratized in 2012 with a view to attaining popular election of the CE in 2017 and of the whole LegCo in 2020. Endorsement from China seemed crucial and determining on the democratic development in Hong Kong. And whether a consensus can be reached among the various political stakeholders in Hong Kong, and the roadmap to implementing universal suffrage for the elections of the CE and the LegCo remain unclear.

Previous studies pointed out that Hong Kong people, along with the people in similar hybrid regimes, may have developed a political fatigue due to their prolonged but futile attempts of attaining a full democracy (Ma 2011). In fact, the ambivalence of Hong Kong people’s commitment to democracy was documented by Lam and Kuan who found that only 37 % of the respondents were very strong or strong supporters of democracy, as compared to other East Asian countries, namely 36.9 % in China, 37.2 % in Taiwan, 58.6 % in South Korea, and 59.8 % in Japan (2008: 28, 205). Similarly, Chu and Huang (2010: 118) categorized only 26.4 % of the Hong Kong respondents in their survey as consistent democrats, who both firmly believe in the principles of a liberal democracy and expressively support it, as compared to 48.8 % in Japan, 31.7 % in South Korea, 28.8 % in Taiwan, 26.5 % in China, and 20.6 % in Singapore. Hong Kong, though not the only East Asian society that exhibits weak and inconsistent support to democracy, is clearly one among the few. It is worth of further analysis for shedding light on the factors that hinder democratic transition in other transitional societies in East Asia.

If democratization is a matter of the people and by the people, it would be important to know how much they support democracy, both in principle and in practice. In the previous ABS, two questions were asked to help determine the extent of DS; namely, first, whether democracy is always preferable to any other form of government, and second, how much democracy the respondents want Hong Kong to achieve. This paper will further investigate into the extent of DS in Hong Kong by analyzing the relevant findings on these two questions.

## Explaining Democratic Support in Hong Kong

As previously stated, this paper examines the relationship between the three factors of EC, AU and NA, with the dependent variables of DS. These three factors are chosen for further analysis in the hypothesized model based on related theories and the empirical observation that all of them are commonly perceived as dominant explanations for DS or the lack of it in Hong Kong. Their comparative significance in affecting DS thus constitutes an interesting area for examination.

### Economic Evaluations and Democratic Support

According to the theories of democratic consolidation, the more economically developed a place is, the higher the people’s democratic aspiration is. Economic development, associated with urbanization, growth of GDP and living standards, and so on, would enhance people’s political expectations of the government towards greater accountability, transparency and democracy (Lipset 1960). In a similar vein, economic decline, such as a decline in income, increases the likelihood of failures of regimes, especially democracies (Przeworski et al. 2000: 109; Diamond 2011: 17–18).

Along with the studies of the importance of objective economic factors on democratic aspiration above-stated, no less influential are a large body of literature on economic voting in which the importance of subjective economic evaluations on the satisfaction with and support for democracy is unraveled. With particular relevance here, these studies, which analyzed Western countries like the United States, found that subjective economic evaluations, including retrospective and prospective evaluation of personal, household and national economic conditions, do affect the level of support for democracy. Specifically, positive subjective economic well being, evaluation and experience will cultivate greater satisfaction with the democratic regime, and thus greater support for democracy. Similarly, more favorable evaluations or perception of the national economy are associated with higher trust in the government’s responsiveness, and thus greater democratic support (Fiorina 1981; Lockerbie 1991).

Importantly, such findings on the relationship between economic evaluations and democratic support not only apply to Western democracies, but also to new democracies. For example, in the study of the parliamentary election in Russia in 1995, Colton (1996) found that voters who held more negative economic evaluations tended to vote for candidates with socialist and nationalist orientations; whereas those who were relatively economically contented had cast their support for reformist candidates. Similarly, in Heslie and Bashkirova’s study on Russia, it was again confirmed that voting behaviour was affected, among other factors, by individual economic experience and perception of the economic future of the nation. Specifically, positive economic evaluations of one’s family and the country, both retrospective and prospective, stood as “independent predictors of evaluations for the democratic reformer” such as Yeltsin (2001: 393). On the contrary, negative expectations about the family’s and the country’s economic future constituted “a rejection of the course of democratic reform” (2001: 394; see also Neundorf’s research on Eastern Europe 2010).

Positive association between economic evaluations and democratic support is also seen in East Asia. The studies of Wu and Chu (2007: 14), and Shyu (2007: 19), both on seven democratic countries, found that positive economic evaluations induced respondents to think that their governments were responsive to the people’s needs, and thus giving support to the incumbent democratic governments. In a similar vein, respondents who disagreed that people in their countries had basic necessities like food, clothes, and shelter tended to endorse democracy less (Wu and Chu 2007: 17).

It would thus be of great interest to examine if the above positive relationship between economic evaluations and democratic support also exists in Hong Kong, or if the relationship is manifested in different manner. As preliminary documented by Chan and Lee (2009: 28–29), negative economic evaluations have contributed to similarly negative evaluations of the Hong Kong, and Chinese, governments, which are not democratically elected, and hence greater democratic aspiration. In this sense, economic evaluations and democratic support are negatively associated in Hong Kong, instead of the positive association found in the countries discussed above.

However, Chan and Lee’s observation may not stand. Previous researches pointed out that Hong Kong people are pragmatic and materialistic who hold negative views about democracy taking democratic development as detrimental to economic development (e.g., Lau and Kuan 1988). When faced with value choices such as the choice between economic development and democracy, the people of Hong Kong would be more inclined to opt for the former (Lam and Kuan 2008). Nevertheless, it is possible that if the economy is perceived by the people as doing well, they will feel more secure and give greater support to democratic development. If this is true, then the association between economic evaluations and democratic support may be positively associated in Hong Kong, instead of the negative association argued by Chan and Lee. Like the Western democracies and new democracies discussed above, more positive economic evaluations will lead to greater democratic support in Hong Kong. Unlike these countries where democratic support is induced by people’s satisfaction with the economic achievements of their democratic governments, in Hong Kong, democratic support is induced by people’s optimism about the economy.

Further examination on the impact of economic evaluations on democratic support in Hong Kong helps understand not only its own arduous road to democracy but also that of other transitional societies where democracy is often depicted as detrimental to economic development.

#### **Hypothesis 1**

The more positive the EC, the greater the DS.

### Authoritarian Alternatives and Democratic Support

In theory, for democratic support to grow, democracy must have gained increasing legitimacy among the people. This means that illiberal and undemocratic values, systems and arrangements, such as elitism, worship of political authority, military rule, strongman rule, rule without alternation, elitist rule, an efficient but undemocratic government, and so on, which are not in line with democratic ones would be rejected by people.

Nevertheless, a previous study on selected Asian countries, not including Hong Kong, on the related subjects (Chang et al. 2007: 72–73) found that the people of these countries felt ambivalent about democracy. They also exhibited lingering support for authoritarianism. Although individual authoritarian alternatives including strongman rule, military rule and single party rule were rejected by the majority of the respondents in the survey, only in South Korea (76.8 %) and Taiwan (69.1 %) did a majority of them rejected all three together. Regarding illiberal and undemocratic arrangements, such as whether the government should decide if certain ideas should be discussed in society, whether judges should accept the views of the executive when they try major cases, and whether the government can disregard the law when the country is facing difficult situations, not all but only a few countries’ respondents displayed general strong disagreements.

Regarding Hong Kong, although a strong majority of the respondents in the 2002 ABS opposed to the authoritarian alternatives listed above, their attitudes towards democracy were ambivalent as earlier stated (Lam and Kuan 2008). Nevertheless, to date, no updated and comprehensive analysis has been conducted on the empirical relationship between pluralist attitudes, detachment from authoritarian alternatives, and democratic support in Hong Kong. Hong Kong, as a semi-democracy, is thus a good case of transitional societies which have been on a bumpy road to democracy. The following analysis attempts to fill this gap in the literature by unraveling the relevance of selected beliefs and attitudes to explaining democratic transition, which will also benefit our understanding of other similar societies.

#### **Hypothesis 2**

The greater the rejection of AU, the greater the DS.

### Nationalism and Democratic Support

Theoretically speaking, the concepts of nationalism and democracy do not necessarily contradict each other, and this explains why there is few, if not nil, research on their mutual influences. Nevertheless, Hong Kong may be unique in this regard due to its truncated histories of being a British colony and widely exposed to universal values on the one hand, and a Chinese city after the political resumption and challenged by contradicting belief systems on the other hand.

Previous researches showed that nationalism in Hong Kong has never been a unified idea. Owing to Britain’s colonial rule of Hong Kong for over a 100 years and the fact that China had turned communist since 1949 and adopted a closed-door policy, Hong Kong people had developed a relatively detached attitude from their motherland, at least before the political resumption in 1997. On the one hand, for most of the time before 1997, the people were largely portrayed as the rootless generations who understood nationalism mainly from a cultural-oriented perspective, signified by their identification with the Chinese tradition and culture, instead of a state-oriented perspective that endorsed the communist state and one-party rule. On the other hand, as Hong Kong became more prosperous and cosmopolitan, the people have gradually acquired relatively liberal, pluralistic and pro-democracy attitudes. Despite the relatively detached attitude from the motherland, state nationalists, meaning those people who felt strong identification with the Chinese communist regime, were not lacking in Hong Kong (Mathews et al. 2008: 10–18).

While the diversity of political beliefs, including the understandings of nationalism, was largely accepted as natural in Hong Kong before the political handover, such diversity has come to be perceived by the Chinese authorities as a threat to achieving national unity in post-handover Hong Kong. In particular, the aspiration for democracy of many Hong Kong people is seen as harmful to the promotion of a state-oriented nationalism which demands total political support for the communist state and one-party rule, and which the Chinese authorities is dedicated to promote. So, although theoretically speaking, the concepts of nationalism and democracy do not necessarily contradict each other, these two ideologies in the political reality of Hong Kong after 1997 have come to represent contrasting political ideals and identity for the people of Hong Kong, and serve different political functions.

Examples of clashes of the two ideologies or identities are ample, especially after Hong Kong’s resumption to China and notably between the state nationalists and the liberal and social democrats. For instance, those who opposed to the legislation of the national security bill in relation to Article 23 of the Basic Law in 2003 were once labeled as unpatriotic. Article 23 authorizes the Hong Kong government to legislate to prohibit subversion against the Beijing government, the theft of state secrets, and political activities by foreign organizations. In 2004, the patriotism debate involved the high profile criticisms of Hong Kong people and leaders of the pro-democracy camp, such as Martin Lee, by Chinese officials and pro-Beijing politicians, as unpatriotic and having “collusion with foreign powers”. In addition, the democracy movement in Hong Kong, since the political resumption, has been obstructed by Beijing and its local representatives who claim that democratization in Hong Kong has to adopt a gradual and consensual approach and observe “China’s situation”; in a word, democratization with Chinese characteristics.

In view of the ongoing conflicts between the ideologies of nationalism and democracy, it would be interesting to further examine their relationship in the context of Hong Kong, which will also fill the gap in the existing literature on the impact of nationalism on democratic support.

#### **Hypothesis 3**

The greater the NA, the lower the DS.

## Analysis: The Three-Factor Model

The present analysis is based on the 2007 ABS on Hong Kong. The data of the survey were collected through a territory-wide face-to-face household survey with Hong Kong Chinese aged 18 or above as its target population. The samples in the survey were prepared by means of a multi-stage design that first selected a sample of 1,813 households from the sampling frame of living quarters provided by the Hong Kong Census and Statistics Department. Interviewers then called at each address and listed all households residing there. Having selected the household, the interviewer was required to list all eligible household members currently aged 18 or over arranged in age order. The respondent was then selected based on a random selection grid (a modified Kish Grid) pre-attached to each address assignment sheet. The interviews were conducted largely from the end of September to November 2007. Altogether 849 cases were successfully completed with a response rate of 46.8 %, yielding a maximum error margin of ±3.36 % at the 95 % confidence level.

### The Measurement Model

In analyzing the reasons for the relatively low DS in Hong Kong, this paper has employed the CFA, which is a theory-driven method for the investigation and confirmation of the relationships between observed measurements and latent factors of a hypothesized theoretical model. The factors of EC, AU and NA are constructed for further analysis due to their importance as dominant explanations in related theories and empirical observations and studies.

As stated, based on the previous ABS, two dependent variables that form the latent variable of DS were selected for analysis in this paper. The question on the first dependent variable explores respondents’ extent of unconditional support for democracy by asking them to choose among the statements of “democracy is always preferable to any other form of government”, “under certain circumstances, an authoritarian government is better than a democracy”, and “for people like me, it is the same whether the government is democratic or not”. For the second dependent variable, respondents were asked to indicate how much democracy they want their country to achieve on a ten-point scale from “1” denoting complete dictatorship to “10” complete democracy.

Table 1 shows the comparative findings on the two questions of selected East Asian countries from the previous ABS. There is no doubt that the people of Hong Kong, along with the people of the East Asian countries in comparison, aspired to actual democracy in their countries. In both the 2002 and 2007 surveys, more than 70 % of the Hong Kong respondents wanted “close to complete democracy” and “complete democracy”, that is, from point 8–10 inclusive. In this regard, Hong Kong excelled in the 2002 survey and was only behind Japan and South Korea in 2007. Nevertheless, on the question of rendering unconditional support to democracy, Hong Kong exhibited the lowest level of support in the 2002 survey, with only 40.4 % of the respondents who agreed that democracy is always preferable to any other form of government, and ranked the second lowest in 2007 with only 43.1 % on the same question. Hong Kong, as one of the few East Asian societies that has exhibited weak and inconsistent support for democracy, is a case worthy of further analysis. Although it is true that the discrepancy between the extent of unconditional support and aspiration for actual democracy was also witnessed in other countries in comparison, Hong Kong has exhibited large and persistent discrepancies in this regard.

**Table 1:** Comparative findings on the dependent variables (in %)

Country	Hong Kong		South Korea		Japan		Taiwan		China	Singapore
Years	2002	2007	2002	2007	2002	2007	2001	2006	2008	2006
(a) Democracy is always preferable	40.4	43.1	49.4	42.7	68.5	63.4	42.8	47.5	53.6	58.9
(b) Democracy for own country (pt 8–10 inclusive)	75.8	70.7	63.2	80.8	74.5	79.3	53.7	63.8	53.9	62.3
(b) − (a)	35.4	27.6	13.8	38.1	6.0	15.9	10.9	16.3	0.3	3.4

% were calculated with missing values included

Table 2 illustrates the overall measurement model. There are altogether 27 observed variables forming three latent variables, namely EC, AU and NA, which will be tested on their relationship with DS. Reverse coding was done on all the related items of EC, AU and NA so that (1) higher scores mean more positive EC, and greater DS, (2) higher scores mean greater rejection of AU, and greater DS, and (3) higher scores mean weaker NA, and greater DS.

**Table 2:**
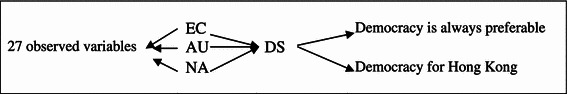
The measurement model

Moreover, with a covariance of 0.53, the two dependent variables form the latent variable of DS. The covariance of the variable of whether democracy is always preferable and DS is 1.49, whereas that of how much democracy for Hong Kong and DS is 0.36 (*t* value = 9.147***). It should be noted that in the structural equation model of LISREL, the hypothesized association between an observed variable and its latent variable, or the loading of an observed variable on its latent variable, is represented by the coefficients above presented. Such hypothesized associations, nevertheless, require significant *t* values (>1.96). Significant *t* values indicate that the hypothesized associations are significant, thus rejecting the null hypothesis of no-effect. A significant level of 0.05 is achieved with a *t* value >1.96, whereas a significant level of 0.01 and 0.001 are achieved with *t* values above 2.58 and 3.29 respectively. In this paper, the hypothesized associations between the two variables and DS have a *t* value >1.96, thus rejecting the null hypothesis of no-effect, and the measurement model for DS can be established.

Altogether nine variables constitute the factor of EC. These variables spread across the scope of our respondents’ evaluations of Hong Kong’s overall economic conditions, personal economic conditions, the government’s capability of solving the economic problems, and preference between economic development and democracy. The variables were chosen based on the existing literature that illustrates the impact of EC on DS.

The relationships among the variables of EC are statistically significant and represent a fairly good fit between the model and the data as presented in Table 3 (*P* value = 0.000, χ^2^ = 195.454, *df* = 27, NFI = 0.83, CFI = 0.85, AGFI = 0.919, RMSEA = 0.086). NFI (normed fit index), AGFI (adjusted goodness of fit index) and CFI (comparative fit index) run from 0 to 1 with a larger value indicating a better model fit. Worthy of note here, a model fit with a value of 0.8 or above is considered acceptable, with controversies though that some scholars regard a higher value of 0.9 or above as the ideal whereas some others do not consider this a golden rule (Bollen 1989; Fan and Sivo, 2005; Greenspoon and Saklofske 1998; Hu and Bentler 1999; Shevlin et al. 2000). RMSEA (root mean square error of approximation) value ranges from 0 to 1 and it is expected that a smaller value means a better model fit. By convention, an acceptable model fit is indicated by a value of 0.08 or less. The variables of the factor of EC are listed in Table 3.

**Table 3:**
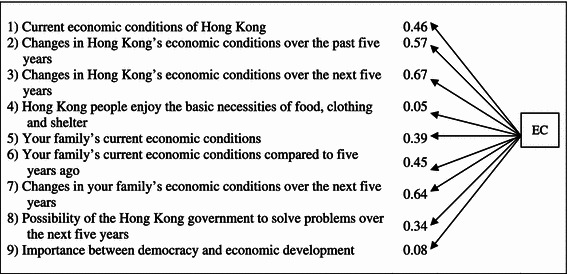
Relationship among the observed variables of economic evaluations (EC)

Regarding the factor of AU, there are 12 variables including pluralist values, attitudes towards paternalistic rulers and arrangements (such as military rule, single-party rule), and preference between authoritarian and democratic governments. The variables of AU were chosen based on the existing literature that has unraveled the concept of authoritarian support as consisting of people’s attitudes towards military rule, a strong executive and its relationship with the legislature and the judiciary, the executive’s role in governing the public opinion and social groups, a paternalistic government, and pluralism.

The relationships among the variables of AU are statistically significant and represent a very good fit between the model and the data as presented in Table 4 (*P* value = 0.000, χ^2^ = 210.696, *df* = 54, NFI = 0.971, CFI = 0.978, AGFI = 0.943, RMSEA = 0.059). The variables are listed in Table 4.

**Table 4:**
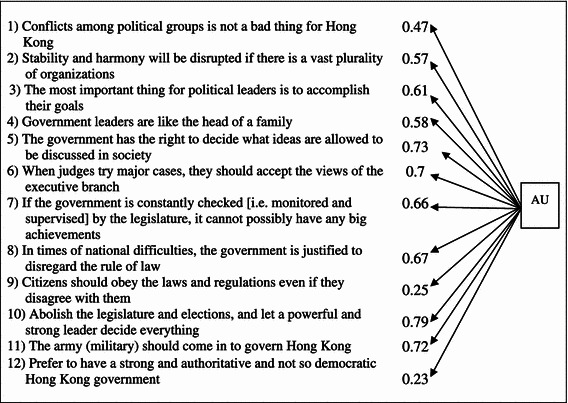
Relationship among the observed variables of authoritarian alternatives (AU)

The factor of NA contains six variables touching on the issues of nationalist values versus individual interests, citizen responsibilities, and national identity. The variables of NA are limited in number due to the original design of the survey questionnaire. Nevertheless, the available variables and findings analyzed here are all relevant and related to citizens’ cognitive beliefs of the ideal relationship between a nation and its people, and their affective and behavioral identification with the Chinese nation, such as whether they feel proud to be Chinese citizens and willing to move and live in other countries.

The relationships among the variables of NA are statistically significant and represent a very good fit between the model and the data as presented in Table 5 (*P* value = 0.000, χ^2^ = 23.341, *df* = 9, NFI = 0.938, CFI = 0.961, AGFI = 0.979, RMSEA = 0.043). The variables are listed in Table 5.

**Table 5:**
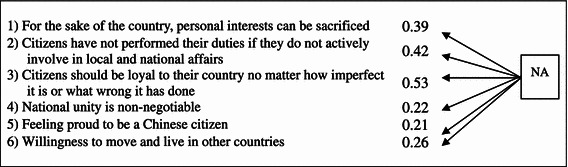
Relationship among the observed variables of nationalism (NA)

Table 6 presents the covariance of the factors of EC, AU and NA. There is a strong covariance between EC and AU (0.461, *P* value = 0.000) and that of EC and NA (0.325, *P* value = 0.000). NA and AU are even more strongly related with a coefficient of covariance of 0.877, *P* value = 0.000.

**Table 6:** Covariance matrix of the three factors

	EC	AU	NA
EC	1		
AU	0.461 (*t* value = 12.328***)	1	
NA	0.325 (*t* value = 6.249***)	0.877 (*t* value = 31.345***)	1

### Findings on the Observed Variables of the Factors

Tables 9, 10 and 11 present the findings on the observed variables of EC, AU and NA (“Appendix”). It is noticed from Table 9 that in general, the respondents gave positive evaluations on Hong Kong’s economic conditions. The majority of them rated Hong Kong’s present economic conditions as very good or good (40.9 %), and its economy over the last 5 years as much or a little better (67.1 %). With regard to the economy 5 years from now, most of the respondents considered that it would become much or a little better (43.6 %). Also, the majority of them strongly agreed or agreed (77.9 %) that Hong Kong people had enjoyed basic necessities.

Similar to their evaluations of Hong Kong’s economy, the respondents also held relatively positive views of their household economic conditions. Concerning their own household economic conditions, the majority of respondents thought it was so so (64.9 %). Most of them perceived their household economic conditions over the last 5 years as about the same (44.4 %) or had become much or a little better (31.6 %). Also, about one-third of them (34.6 %) thought their household economic conditions 5 years from now would become much or a little better.

Nevertheless, there is a divided opinion on whether the government would be capable of solving the most important problems, including economic problems, with 33.2 % claiming highly likely or likely and 43.4 % very unlikely or unlikely. Despite the respondents’ positive evaluations of Hong Kong’s economy and their household economic conditions, they did not hold a positive view of democracy. The majority of them considered economic development as definitely or somewhat more important than democracy (71.1 %).

Table 10 presents the findings on the observed variables of AU. It is shown that a good proportion of the respondents were rather intolerant of the plurality of organizations and its possible consequences. Although 56 % of them strongly agreed or agreed that conflict among political groups is not a bad thing for Hong Kong, 32 % strongly disagreed or disagreed. Also, 47.9 % of them strongly agreed or agreed that social stability and harmony would be disrupted if there was a vast plurality of groups.

As regards the roles and responsibilities of political leaders and the government, the majority strongly disagreed or disagreed (71.6 %) that the most important thing for political leaders was to accomplish their goals even if they had to ignore the established procedures. Also, 62.7 % strongly disagreed or disagreed that government leaders were like the head of a family, and we should all follow their decisions. Nevertheless, their attitudes were not completely liberal and egalitarian. Regarding whether the government had the authority to decide if certain ideas were allowed to be discussed in society, the opinion was quite divided with 50.4 % who strongly disagreed or disagreed, and 36.8 % strongly agreed or agreed. Similarly, 52.7 % strongly disagreed or disagreed that judges should accept the views of the executive branch when they try major cases, whereas 34.1 % strongly agreed or agreed. Furthermore, 43.2 % strongly disagreed or disagreed that if the government was constantly checked by the legislature, it could not possibly have any big accomplishments, whereas 43.5 % strongly agreed or agreed.

Regarding the respondents’ views of selected authoritarian options, they appeared to be ambivalent. On the statement that the government was justified to disregard the law to deal with the situation in times of national difficulties, 69.4 % of the respondents strongly disagreed or disagreed. And there were 78.8 % of the respondents who strongly disagreed or disagreed that we should abolish the legislature and elections and have a strong leader to decide things. On whether the military should govern Hong Kong, 87.6 % of them strongly disagreed or disagreed. Nevertheless, on whether the people should obey the laws and regulations even if they disapproved of them, 85.6 % strongly agreed or agreed. Lastly, despite the general disagreements with the authoritarian options stated, the respondents were again divided on whether they wanted a government which was strong and authoritative but not very democratic (38.9 %) or a government which was not so strong and authoritative but democratic (35 %).

Table 11 presents the findings on the observed variables of NA. The respondents were found to be relatively divided on their attitudes towards citizen duties. Regarding whether the people should be prepared to sacrifice their personal interests for the sake of the country, 42.6 % of them strongly agreed or agreed while 40.6 % strongly disagreed or disagreed. Also, 37.6 % of them strongly agreed or agreed that a citizen who did not actively participate in local and national affairs was not performing his/her duties, whereas 55.2 % strongly disagreed or disagreed.

The respondents seemed to maintain a strong pride being part of their country and place of residence. Altogether 62 % of them strongly agreed or agreed that a citizen should always remain loyal to his/her country, no matter how imperfect it is. There were 59.2 % of them who considered that it was essential for the country to remain one nation; and 82.6 % of the respondents felt very or somewhat proud to be a Chinese citizen. Lastly, only 28.3 % of them were very willing or willing to go and live in another country if given the chance.

### Results of Confirmatory Factor Analysis

Table 7 presents the results of the overall structural model which is statistically significant and a very good fit (*P* value = 0.000, χ^2^ = 1,144.191, *df* = 371, NFI = 0.931, CFI = 0.953, AGFI = 0.9, RMSEA = 0.049). All the hypothesized paths are also significant at the 0.05 level or above. AU is related to DS with an estimate of 0.97, EC to DS with an estimate of 0.22, and NA to DS with an estimate of 0.07. The results indicate that, in the case of Hong Kong, people’s pluralist attitudes and attitudes in relation to AU affect their support for democracy the most, next is EC and then NA. The squared multiple correlations for structural equations of DS is 0.685, meaning that the model can explain a good proportion of the data of over 68 %.

**Table 7:**
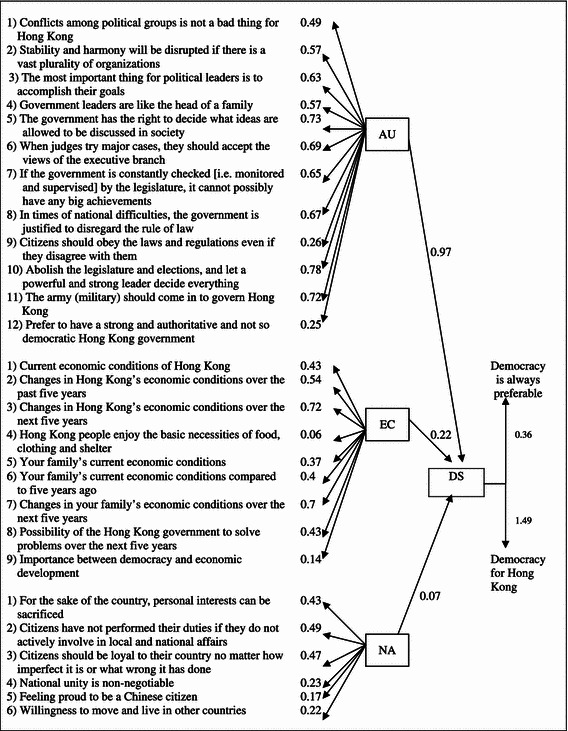
Results of model fit

The hypothesized model, as a very good fit model, indicates that Hong Kong people’s relatively low DS, in terms of their unconditional support for democracy and the degree of democracy they want for Hong Kong, can be well explained by the three factors of EC, AU and NA in combination. The factors have various extent of impact on DS, in principle and in practice, with AU being the strongest, followed by EC and then NA. The degree to which people are supportive of democracy in principle and in practice is affected by such competing values and choices.

Hypothesis 1 is confirmed that the more positive one’s EC is, the greater his/her DS. The impact of EC on DS is positive and evident with an estimate of 0.22. The favorable economic experiences of the people of Hong Kong had not increased their identification with the undemocratic governance of the government nor decreased their levels of DS, which is interestingly different from Chan and Lee’s findings (2009). Rather, as the Hong Kong people become more optimistic of the economic condition, they are also more prepared to give greater support to Hong Kong’s democratic development.

The observed variables of EC include the evaluation of Hong Kong’s overall economic condition, personal economic condition, the government’s capability of solving the economic problems, and preference between economic development and democracy. Among all the observed variables, people’s evaluation of the Hong Kong economy, and personal economic condition 5 years from now have particularly strong relationship with EC, at 0.72 and 0.7 respectively. This implies that people’s perception of their economic future constituted the more important types of EC which in turn affects DS. This is consistent with the findings of the previous researches on the significance of prospective economic evaluations on DS.

Hypothesis 2, which states that the greater the rejection of AU, the greater the DS, is also confirmed in the analysis. Worthy of note, AU has particularly strong impact on DS with a positive estimate of 0.97. These findings also testify to the analysis of the previous study on selected Asian countries that support for democracy is highly related to the rejection of authoritarian values and alternatives and importantly, as illustrated above, to pluralism.

The observed variables of AU include pluralist values, attitudes towards paternalistic rulers and arrangements (such as military rule, single-party rule), and preference between authoritarian and democratic governments. Among all, the variables of whether we should abolish the legislature and elections, and let a strong leader decide everything (0.78), whether the military should come in to govern Hong Kong (0.72), whether the government has the right to decide what ideas are allowed to be discussed in society (0.73), and whether when judges try major cases, they should accept the views of the executive branch (0.69) are found to be more strongly associated with AU, and hence with DS. All of these items indicate the respondents’ attitudes towards certain concrete political arrangements of how the executive should rule and relate to other organs of the government such as the judiciary, the legislature and the military. Future research may further compare if people’s attitudes in these regards have greater impact on their DS than their other political attitudes such as extent of pluralism and political tolerance.

Regarding Hypothesis 3, NA, with a positive estimate of only 0.07, has little impact on DS. However, since the overall hypothesized model is statistically significant and a very good fit, Hypothesis 3 can still be confirmed. This means that the more nationalistic a person is, the less his/her DS. Along with this, the variables of NA itself constitute a good fit model, and NA is significantly related to EC and AU (the other two latent factors). So, despite of the small coefficient, NA plays a part, in combination with EC and AU, in explaining DS.

The observed variables of NA include issues of nationalist values versus individual interests, citizen responsibility, and national identity. In particular, the variables on citizen duties are associated more strongly with NA, comparing to other variables, and hence with DS as well. These variables include whether personal interests can be sacrificed for the sake of the country (0.43), whether citizens who do not actively involve in local and national affairs are not performing their duties (0.49), and whether citizens should be loyal to their country no matter how imperfect it is or what wrong it has done (0.47). These may imply that, in the context of Hong Kong, how nationalistic people are is greatly related to the extent of their unconditional regard for the national interests. And those who have greater unconditional regard for the country are less supportive of democracy. These findings coincide with our observation of the contradiction between NA and DS in Hong Kong. Nevertheless, in view of the low estimate of NA on DS, future research may include more relevant observed variables on the perceptions of citizen responsibility in testing the relationships.

### Demographic Variables and the Model

As often, demographic variables, including gender and age, may affect political values and DS. Thus, analyses of the factor loading invariance of the three-factor measurement model established in this article were carried out to evaluate whether the model is invariant across gender and age.

These assessments were conducted through a multi-group analysis in LISREL that re-analyzes the data, and investigates the similarities and differences among different sub-samples defined by gender and age.^2^ The idea is to compare the model fit when we estimate the individual model parameters separately for the different sub-samples to the fit. Worthy of note, there are different approaches to invariance testing. This article adopts a model-based approach to invariance testing using fit indices, such as CFI as cut-off scores, to examine whether the invariance hypothesis be rejected. Some scholars, however, would use χ^2^ tests or a mixed approach of both fit indices and χ^2^ tests to determine the lack of fit. The debate on these methodologies is beyond the scope of this article and thus will not be dealt with here (Hirschfeld and Brown 2009: 32; Little 1997).

Table 8 compares the goodness of fit statistics of the present model and other models. With the variable of gender included, the model remains a very good fit, with both the sub-samples of female and male achieving good indices. With the variable of age included, the goodness of fit of the model also remains satisfactory, with non-substantial decreases in a few indices though, such as a lower value of NFI. Overall, the analysis illustrates that the present three-factor model is relatively invariant across the sub-samples of gender and age, and thus is justified.

**Table 8:** Comparison of goodness of fit statistics of the models

	Present model	Gender		Age	
		Female	Male	≤39	Above 39
χ^2^	1,144.191	752.392	803.9	574.77	940.96
*df*	371	371	371	371	371
*P* value	0.000	0.000	0.000	0.000	0.000
NFI	0.931	0.933	0.842	0.716	0.927
CFI	0.953	0.965	0.909	0.874	0.956
AGFI	0.9	0.881	0.853	0.85	0.879
RMSEA	0.049	0.047	0.055	0.045	0.052

Tables 12, 13 (“Appendix”) indicate the factor loadings of the invariance tests of the variables of gender and age on the observed variables of EC, AU and NA as well as DS. Almost all of the factor loadings are statistically significant with only a few exceptions.

## Discussion

Overall speaking, the hypothesized model established in this paper can be further tested and applied in analyzing DS in other transitional countries or Hong Kong in different periods of time. The findings further shed light on the possibilities of increasing DS in Hong Kong and elsewhere. Importantly, greater DS may be garnered through education that consolidates people’s acceptance of pluralistic values and rejection of authoritarian alternatives. Along with this, people’s optimism about the economy will also probably increase their DS. And any government attempts, especially in transitional societies, to cultivate fear among the people that the economy is declining or will decline, or the trigger of such fear in the people by bad economic conditions, will likely be harmful to the consolidation of DS. With regard to NA and DS in Hong Kong, there is the likelihood that the more nationalistic the people are, the less supportive of democracy they will be. Nevertheless, this area deserves further research as the meaning of being nationalistic is open to various interpretations. The nationalism of the Hong Kong people may tend to be more culturally-oriented instead of state-oriented, and thus less indicative of the people’s total support of the Chinese communist regime. If so, having a strong national identity may not necessarily imply being less supportive of democracy for Hong Kong.

Methodologically, this paper illustrates the usefulness of CFA in political values research, especially in examining how the values in analysis produce certain attitudinal and behavioral outcomes, for example, DS, in combined efforts. Importantly, the method helps assess the comparative significance of a multiplicity of values and factors selected for analysis in a hypothesized model, which will facilitate the making of informed choices on the directions of future research.

Regarding the limitations of the study, in the present model, NA has a low estimate on DS. This may be further investigated in future research by including more relevant observed variables for testing. Moreover, the present model, with its focus on the relationships between the three factors (EC, AU and NA) and DS, may be further developed by examining whether the model is also invariant across different sub-samples defined by other socio-economic characteristics and social values, e.g., familial values, of the respondents.

## Conclusion

This paper examines the reasons for the relatively low DS in Hong Kong in the context of competing values and choices based on the previous ABS. In so doing, it establishes a three-factor theoretical model that includes survey attitudinal statements related to EC, AU and NA on DS. Using CFA of LISREL, the above analysis shows that the hypothesized model is a very good fit. The Hong Kong people’s relatively low DS, in terms of their unconditional support for democracy and the degree of democracy they want for Hong Kong, can be well explained by the three factors: EC, AU and NA in combination. The factors have various extent of impact on DS, with AU being the strongest, followed by EC and then NA. The paper contributes empirically by unraveling the relative importance of the range of values and choices affecting Hong Kong people’s support for democracy. Theoretically, the model established in the paper can be further tested and applied in analyzing DS in other transitional countries and other periods of Hong Kong. Methodologically, the paper illustrates the usefulness of CFA in assessing the comparative significance of a wide spectrum of values and factors in political values research.

## Appendix

See Tables 9, 10, 11, 12 and 13.

**Table 9:** Findings on the observed variables of EC (in %)

	5 Very good	4 Good	3 So so	2 Bad	1 Very bad
(1) Current economic conditions of Hong Kong	2.1	38.8	44.6	11.3	2.1
(5) Your family’s current economic conditions	0.5	19	64.9	12.7	2.1

	5 Much better	4 A little better	3 About the same	2 A little worse	1 Much worse
(2) Changes in Hong Kong’s economic conditions over the past 5 years	10.1	57	15.9	12.7	2
(3) Changes in Hong Kong’s economic conditions over the next 5 years	4.5	39.1	24.9	11.3	1.2
(6) Your family’s current economic conditions compared to 5 years ago	2.4	29.2	44.4	20.5	2.9
(7) Changes in your family’s economic conditions over the next 5 years	4.2	30.4	35.7	12	0.5

	5 D definitely more important	4 D more important	3 About the same	2 ED more important	1 ED definitely more important
(9) Importance between democracy and economic development	1.8	7.9	16.3	42.4	28.7

	4 Strongly agree	3 Agree	2 Disagree	1 Strongly disagree
(4) Hong Kong people enjoy the basic necessities of food, clothing and shelter	6.2	71.7	18.7	1.3

	4 Very likely	3 Likely	2 Unlikely	1 Very unlikely
(8) Possibility of the Hong Kong government to solve problems over the next 5 years	1.5	31.7	33.3	10.1

Total number of cases is 849; % were calculated with missing values included

*D* democracy, *ED* economic development

**Table 10:** Findings on the observed variables of AU (in %)

	4 Strongly agree	3 Agree	2 Disagree	1 Strongly disagree
(1) Conflicts among political groups is not a bad thing for Hong Kong	2.1	53.9	28.9	3.1

	4 Strongly disagree	3 Disagree	2 Agree	1 Strongly agree
(2) Stability and harmony will be disrupted if there is a vast plurality of organizations	2.5	41.5	45.3	2.6
(3) The most important thing for political leaders is to accomplish their goals	11.8	59.8	17.6	0.8
(4) Government leaders are like the head of a family	4.2	58.5	28.3	0.9
(5) The government has the right to decide what ideas are allowed to be discussed in society	4.9	45.5	35.9	0.9
(6) When judges try major cases, they should accept the views of the executive branch	6.5	46.2	32.6	1.5
(7) If the government is constantly checked (i.e. monitored and supervised) by the legislature, it cannot possibly have any big achievements	1.9	41.3	41	2.5
(8) In times of national difficulties, the government is justified to disregard the rule of law	9	60.4	17.4	0.6
(9) Citizens should obey the laws and regulations even if they disagree with them	0.1	8.8	78.3	7.3
(10) Abolish the legislature and elections, and let a powerful and strong leader decide everything	16.8	62	10.4	0.5
(11) The army (military) should come in to govern Hong Kong	37.7	49.9	4.1	0.4

	2 Not strong and authoritative but relatively more democratic	1 Strong and authoritative but not so democratic
(12) Prefer to have a strong and authoritative and not so democratic Hong Kong government	35	38.9

Total number of cases is 849; % were calculated with missing values included

**Table 11:** Findings on the questions of NA (in %)

	4 Strongly disagree	3 Disagree	2 Agree	1 Strongly agree
(1) For the sake of the country, personal interests can be sacrificed	2.8	37.8	40.2	2.4
(2) Citizens have not performed their duties if they do not actively involve in local and national affairs	2.2	53	35	2.6
(3) Citizens should be loyal to their country no matter how imperfect it is or what wrong it has done	1.4	27.6	57.4	4.6

	4 Not proud at all	3 Not very proud	2 Somewhat proud	1 Very proud
(5) Feeling proud to be a Chinese citizen	0.8	8.5	67.8	14.8

	4 Very willing	3 Willing	2 Not willing	1 Not willing at all
(6) Willingness to move and live in other countries	3.4	24.9	52.9	12.7

	2 Independence should be granted to those regions that opt for independence	1 National unity is non-negotiable
(4) National unity is non-negotiable	21.4	59.2

Total number of cases is 849; % were calculated with missing values included

**Table 12:** Factor loadings of the invariance tests of gender (female and male)

Lambda X	Sub-samples	
	Female	Male
EC		
(1) Current economic conditions of Hong Kong	0.381	0.485
(2) Changes in Hong Kong’s economic conditions over the past 5 years	0.523	0.564
(3) Changes in Hong Kong’s economic conditions over the next 5 years	0.689	0.754
(4) Your family’s current economic conditions	0.411	0.333
(5) Your family’s current economic conditions compared to 5 years ago	0.462	0.340
(6) Changes in your family’s economic conditions over the next 5 years	0.662	0.743
(7) Possibility of the Hong Kong government to solve problems over the next 5 years	0.441	0.408
(8) Hong Kong people enjoy the basic necessities of food, clothing and shelter	*0.051*	0.071
(9) Importance between democracy and economic development	0.140	0.121
AU		
(1) Conflicts among political groups is not a bad thing for Hong Kong	0.525	0.427
(2) The most important thing for political leaders is to accomplish their goals	0.718	0.526
(3) Prefer to have a strong and authoritative and not so democratic Hong Kong government	0.302	0.155
(4) Abolish the legislature and elections, and let a powerful and strong leader decide everything	0.873	0.620
(5) The army (military) should come in to govern Hong Kong	0.827	0.543
(6) Government leaders are like the head of a family	0.629	0.484
(7) The government has the right to decide what ideas are allowed to be discussed in society	0.765	0.667
(8) Stability and harmony will be disrupted if there is a vast plurality of organizations	0.624	0.482
(9) When judges try major cases, they should accept the views of the executive branch	0.759	0.570
(10) In times of national difficulties, the government is justified to disregard the rule of law	0.735	0.530
(11) If the government is constantly checked (i.e. monitored and supervised) by the legislature, it cannot possibly have any big achievements	0.787	0.485
(12) Citizens should obey the laws and regulations even if they disagree with them	0.315	0.176
NA		
(1) For the sake of the country, personal interests can be sacrificed	0.469	0.372
(2) National unity is non-negotiable	0.313	0.085
(3) Citizens have not performed their duties if they do not actively involve in local and national affairs	0.530	0.420
(4) Citizens should be loyal to their country no matter how imperfect it is or what wrong it has done	0.480	0.492
(5) Feeling proud to be a Chinese citizen	0.173	0.198
(6) Willingness to move and live in other countries	0.199	0.265
Lambda Y (DS)		
Democracy for Hong Kong	1.831	1.035
Democracy is always preferable	0.426	0.230

All the factor loadings are significant at the 0.05 level or above except EC(8) (*t* value <1.96) in the female sub-sample

**Table 13:** Factor loadings of the invariance tests of age (aged 39 and below, and above 39)

Lambda X	Sub-samples	
	≤39	Above 39
EC		
(1) Current economic conditions of Hong Kong	0.269	0.472
(2) Changes in Hong Kong’s economic conditions over the past 5 years	0.441	0.568
(3) Changes in Hong Kong’s economic conditions over the next 5 years	0.562	0.747
(4) Your family’s current economic conditions	0.335	0.404
(5) Your family’s current economic conditions compared to 5 years ago	0.410	0.397
(6) Changes in your family’s economic conditions over the next 5 years	0.529	0.651
(7) Possibility of the Hong Kong government to solve problems over the next 5 years	0.178	0.485
(8) Hong Kong people enjoy the basic necessities of food, clothing and shelter	*0.023*	0.085
(9) Importance between democracy and economic development	*0.115*	0.121
AU		
(1) Conflicts among political groups is not a bad thing for Hong Kong	0.212	0.568
(2) The most important thing for political leaders is to accomplish their goals	0.380	0.715
(3) Prefer to have a strong and authoritative and not so democratic Hong Kong government	0.120	0.283
(4) Abolish the legislature and elections, and let a powerful and strong leader decide everything	0.470	0.854
(5) The army (military) should come in to govern Hong Kong	0.370	0.786
(6) Government leaders are like the head of a family	0.389	0.610
(7) The government has the right to decide what ideas are allowed to be discussed in society	0.443	0.774
(8) Stability and harmony will be disrupted if there is a vast plurality of organizations	0.514	0.572
(9) When judges try major cases, they should accept the views of the executive branch	0.432	0.762
(10) If the government is constantly checked (i.e. monitored and supervised) by the legislature, it cannot possibly have any big achievements	0.505	0.701
(11) In times of national difficulties, the government is justified to disregard the rule of law	0.343	0.758
(12) Citizens should obey the laws and regulations even if they disagree with them	0.165	0.286
NA		
(1) For the sake of the country, personal interests can be sacrificed	0.310	0.440
(2) National unity is non-negotiable	0.159	0.251
(3) Citizens have not performed their duties if they do not actively involve in local and national affairs	0.274	0.515
(4) Citizens should be loyal to their country no matter how imperfect it is or what wrong it has done	0.494	0.496
(5) Feeling proud to be a Chinese citizen	0.254	0.160
(6) Willingness to move and live in other countries	0.285	0.159
Lambda Y (DS)		
Democracy for Hong Kong	0.645	1.666
Democracy is always preferable	0.281	0.395

All the factor loadings are significant at the 0.05 level or above except EC(8) and EC(9) (*t* values <1.96) in the ≤39 sub-sample

## Footnotes

1 The author thanks Richie Chu for his excellent research assistance. Also, data analyzed in this paper were collected by the Asian Barometer Project (2005–2008), which was co-directed by Professors Fu Hu and Yun-han Chu and received major funding support from Taiwan’s Ministry of Education, Academia Sinica and National Taiwan University. The Asian Barometer Project Office (www.asianbarometer.org) is solely responsible for the data distribution. The author appreciates the assistance in providing data by the institutes and individuals aforementioned. The views expressed herein are the author’s own.

2 In the following model analysis with the variable of gender included, the sample was divided into the sub-samples of male and female. In the same vein, in the model analysis with the variable of age included, the sample was divided into the sub-samples of those aged 39 and below, and those above 39. For the parsimony of analysis, only two sub-samples were categorized in the analysis of age.
